# cAMP signaling of *Bordetella* adenylate cyclase toxin blocks M-CSF triggered upregulation of iron acquisition receptors on differentiating CD14^+^ monocytes

**DOI:** 10.1128/msphere.00407-24

**Published:** 2024-07-30

**Authors:** Jawid Nazir Ahmad, Peter Sebo

**Affiliations:** 1 Laboratory of Molecular Biology of Bacterial Pathogens, Institute of Microbiology of the Czech Academy of Sciences, Prague, Czechia; University of Kentucky College of Medicine, Lexington, Kentucky, USA

**Keywords:** *Bordetella pertussis*, adenylate cyclase toxin, monocytes, macrophages, differentiation, cyclic AMP, iron acquisition

## Abstract

*Bordetella pertussis* infects the upper airways of humans and disarms host defense by the potent immuno-subversive activities of its pertussis (PT) and adenylate cyclase (CyaA) toxins. CyaA action near-instantly ablates the bactericidal activities of sentinel CR3-expressing myeloid phagocytes by hijacking cellular signaling pathways through the unregulated production of cAMP. Moreover, CyaA-elicited cAMP signaling also inhibits the macrophage colony-stimulating factor (M-CSF)-induced differentiation of incoming inflammatory monocytes into bactericidal macrophages. We show that CyaA/cAMP signaling via protein kinase A (PKA) downregulates the M-CSF-elicited expression of monocyte receptors for transferrin (CD71) and hemoglobin-haptoglobin (CD163), as well as the expression of heme oxygenase-1 (HO-1) involved in iron liberation from internalized heme. The impact of CyaA action on CD71 and CD163 levels in differentiating monocytes is largely alleviated by the histone deacetylase inhibitor trichostatin A (TSA), indicating that CyaA/cAMP signaling triggers epigenetic silencing of genes for micronutrient acquisition receptors. These results suggest a new mechanism by which *B. pertussis* evades host sentinel phagocytes to achieve proliferation on airway mucosa.

**IMPORTANCE:**

To establish a productive infection of the nasopharyngeal mucosa and proliferate to sufficiently high numbers that trigger rhinitis and aerosol-mediated transmission, the pertussis agent *Bordetella pertussis* deploys several immunosuppressive protein toxins that compromise the sentinel functions of mucosa patrolling phagocytes. We show that cAMP signaling elicited by very low concentrations (22 pM) of *Bordetella* adenylate cyclase toxin downregulates the iron acquisition systems of CD14^+^ monocytes. The resulting iron deprivation of iron, a key micronutrient, then represents an additional aspect of CyaA toxin action involved in the inhibition of differentiation of monocytes into the enlarged bactericidal macrophage cells. This corroborates the newly discovered paradigm of host defense evasion mechanisms employed by bacterial pathogens, where manipulation of cellular cAMP levels blocks monocyte to macrophage transition and replenishment of exhausted phagocytes, thereby contributing to the formation of a safe niche for pathogen proliferation and dissemination.

## INTRODUCTION

The “classical” *Bordetella* species *B. bronchiseptica*, *B. pertussis,* and *B. parapertussis* colonize the upper airways of various mammals, with *B. pertussis* and occasionally also *B. parapertussis*_hu_ causing the highly contagious human respiratory infectious disease known as pertussis, or whooping cough (1). *B. pertussis* has to overcome the innate immune defense at host respiratory mucosa and proliferate to sufficiently high numbers to elicit a catarrh that will force the host to sneeze, cough, and transmit the pathogen by aerosol droplets. To proliferate, *B. pertussis* adheres to ciliated epithelial cells of the upper airways, deploying an array of adhesins, complement resistance factors and immuno-subversive protein toxins, which suppress and hijack the host’s innate and adaptive immune defense mechanisms (2). A prominent role in immune evasion is played by the notoriously known pertussis toxin (PT) that is produced only by *B. pertussis* and accounts for the critical hyperleukocytosis and pertussis pneumonia development in unvaccinated infants (3–6). Another potently immunosuppressive toxin is the adenylate cyclase toxin-hemolysin (CyaA, ACT, or AC-Hly) that is produced by all *Bordetella* species pathogenic to mammals and appears to play a key role in the early phases of subversion of host immune defense by *Bordetella* species (7, 8). CyaA targets the complement receptor 3 (CR3), the heterodimeric α_M_β_2_ integrin known also as CD11b/CD18 or Mac-1, which is abundant on sentinel myeloid phagocytes, such as macrophages, monocytes, dendritic cells, and neutrophils (9, 10). Upon binding to CR3 the CyaA toxin penetrates the plasma membrane of phagocytic cells and translocates its adenylyl cyclase (AC) domain into their cytosol. The AC is then activated by cytosolic calmodulin to a highly active AC enzyme that catalyzes massive and unregulated conversion of cytosolic ATP to the key second messenger molecule, cAMP (11, 12). Signaling of CyaA-produced cAMP then hijacks various signaling pathways of CR3-expressing myeloid cells and dampens the host immune responses (13–19).

The airway patrolling macrophages along with chemo-attracted neutrophils and antimicrobial peptides and proteins form the innate immune barrier that protects the airway mucosa from infection. Recently, we showed that the CyaA toxin already at picomolar concentrations provokes de-differentiation of primary human alveolar macrophages to monocyte-like cells and impedes the M-CSF-driven transition of circulating inflammatory monocytes into macrophages (13). Since the M-CSF cytokine is secreted by various cell types, such as respiratory epithelial cells, endothelial cells, fibroblasts, and macrophages, the M-CSF levels regulate the pro-differentiating milieu of the mucosa on which the macrophage pool can be replenished by incoming monocytes (20–22).

Another important factor modulating monocyte differentiation is iron availability, as iron atoms are particularly important for vital cellular functions, including oxygen sensing, mitochondrial respiration, signaling, metabolism, DNA synthesis, etc. (23). In response to the pro-differentiating signals emanating from the M-CSF receptor, the monocytes require increased iron supply in order to acquire the macrophage-defining features, such as the increased cell size, increased numbers of mitochondria and expansion of complex organelles involved in bactericidal functions. Limited iron supply thus promotes cell cycle arrest and apoptosis, thereby hampering the ability of monocytes to acquire the macrophage phenotype (24–29).

Ferric ions (Fe^3+^) are largely imported into cells in complex with transferrin (Tf) bound to the transferrin receptor CD71 (TfR1) (24, 30). The Tf-Fe_2_/CD71 complex is next internalized into acidified endosomes (31), where the transferrin-bound Fe^3+^ is reduced to Fe^2+^ ions that are transported into cell cytosol through divalent metal transporter 1 (32). Another important source of iron for the differentiating monocytes at the mucosal sites is the haptoglobin-bound heme (iron-containing tetrapyrrole), which leaks onto the mucosal surface with plasma components during extravasation of phagocytes into the infected tissue (33–38). Haptoglobin binds free hemoglobin and facilitates its uptake into monocytes and macrophages through the high-affinity scavenger receptor CD163 of the hemoglobin-haptoglobin (Hb-Hp) complex (39). Hb-Hp uptake through CD163 then drives differentiation of macrophages (40) and in case of excess hemoglobin, even the free Hb can be taken up by macrophages through CD163 (41). The imported heme is intracellularly degraded by the *Hmox1*-encoded heme oxygenase-1 (HO-1) enzyme to carbon monoxide (CO), biliverdin, and free iron. HO-1 overexpression has thus been associated with increased intracellular iron availability (42), whereas the unused intracellular iron is exported by ferroportin (Slc40a1, FPN), or stored within ferritin particles (43).

Since CyaA blocks monocyte differentiation into macrophages, we examined if cAMP signaling resulting from CyaA toxin action impairs iron acquisition by differentiating monocytes and we demonstrate that CyaA through its cAMP-elevating activity blocks M-CSF-driven upregulation of expression of the iron acquisition receptors CD71 and CD163.

## MATERIALS AND METHODS

### Antibodies and reagents

PE-Cy7-labeled anti-CD163 (GHI/61) and Alexa647-labeled anti-CD71 (MEM-75) antibodies were purchased from Exbio (Czech Republic). HO-1 polyclonal antibody from Enzo Life Sciences (Cat. number ADI-SPA-896), SLCO2B1 polyclonal antibody from Novus biologicals (Cat. number NBP1-59811). RNA blue was purchased from TopBio (Czech Republic), recombinant human M-CSF from Peprotech (Cat. number 300-25), and human CD14 MicroBeads from Miltenyi Biotec (Cat. number 130-050-201) . Albumin fraction V was purchased from Carl Roth (Cat. number 8076). Rp-8-CPT-cAMPS was purchased from Biolog (Cat. number C011), antibiotic antimycotic solution (100×), Dulbecco’s modified Eagle’s medium (DMEM) (Cat. number D6429), FeCl_2_ (Cat. number 220299), 8-Hydroxyquinoline (Cat. number H6878), and Protoporphyrin IX cobalt chloride (Cat. number C1900) were purchased from Sigma-Aldrich. Micro BCA protein assay kit and High-Capacity cDNA Reverse Transcription Kit (Cat. number 4368814) were purchased from Thermo Scientific (Cat. number 23235). 5× HOT FIREPol EvaGreen qPCR Mix Plus from Solis BioDyne (Cat. number 08-24-00020) and DNase I (Cat. number M0303) were from New England Biolabs. Calcein-AM was from Biolegend (Cat. number 425201), and trichostatin A was from MedChemExpress (Cat. number HY-15144).

### Monocyte separation and analysis of monocyte differentiation state

Blood leukopaks from anonymous healthy donors were purchased at the Thomayer hospital in Prague and were utilized to isolate the CD14^+^ cells from human peripheral blood mononuclear cells (PBMCs) using CD14 MicroBeads (Miltenyi Biotec). The separation of CD14^+^ cells was performed as per the manufacturer’s instructions by using magnetic-activated cell sorting (MACS), reaching a purity of CD14^+^ cells higher than 90% of living cells, as determined by flow cytometry. The CD14^+^ monocytes were used in experiments as previously described (13). Briefly, the monocytes in DMEM containing 20 ng/mL M-CSF and 10% FCS were either exposed to 4 ng/mL of CyaA [with or without 0.5 µM protoporphyrin IX cobalt chloride (CoPP)] or (with or without indicated concentrations of FeCl_2_) or to 4 ng/mL of CyaA-AC^−^. Macrophage differentiation markers were determined after 5 days of culture by flow cytometry with the use of specific fluorophore-labeled antibodies.

### *B. pertussis* strains and infection conditions

*B. pertussis* mutants derived from the Tohama I strain (Institute Pasteur collection, CIP 81.32) were constructed and cultured as previously described (13). The CD14^+^ monocytes were infected with *B. pertussis* wild type or mutant bacteria at a multiplicity of infection (MOI) of 2 bacteria per monocyte (2:1). Briefly, bacterial suspensions were diluted 1:10 in DMEM, and 2 × 10^6^ CFU were added to 10^6^ CD14^+^ monocytes in 1 mL of DMEM with 10% FCS and 20 ng/mL of M-CSF. The bacteria were allowed to infect the monocytes for 12 h before being killed by antibiotics (50 µg/mL polymyxin B plus 50 µg/mL kanamycin) for 12 h. Cells were cultured for an additional 5 days in DMEM with 10% FCS and 20 ng/mL M-CSF, with repeated replacement of half of the medium every 24 h. After 5 days the expression levels of CD71 and CD163 on infected cells were analyzed by flow cytometry, as previously described (13).

### CyaA and CyaA-AC^−^ purification

CyaA and CyaA-AC^−^ were produced in *E. coli* strain XL1-Blue (Stratagene, La Jolla, CA) expressing *cyaC* from the pT7CACT1-derived plasmids (44). Proteins were purified by a combination of ion-exchange chromatography on DEAE-Sepharose and hydrophobic chromatography on Phenyl-Sepharose, as described (44). Endotoxin-free CyaA and CyaA-AC^−^ proteins were obtained by washing the CyaA-loaded resin with 60% isopropanol (45). The purified protein preparations contained less than 0.1 endotoxin units of lipopolysaccharide per 1 µg of protein, as determined by a chromogenic limulus amebocyte lysate assay kit (QCL-1000; Lonza, Walkersville, MD). The CyaA toxin and CyaA-AC^−^ toxoid stocks (1 mg/mL) were kept frozen at −20°C in TUC buffer (Tris 20 mM, Urea 8 M, Calcium 2 mM, pH 7.4). Prior to assay, the stocks were diluted 250-fold in TUC to generate working stocks of 4 µg CyaA/mL, of which 1 µL was added into 1 mL of cell suspension in DMEM (TUC dilution 1:1,000) to reach the final toxin/toxoid concentration of 4 ng/mL (22.5 pM).

### Immunoblotting

The cells were lysed with 10 mM Tris (pH 7.4), 140 mM NaCl, 1 mM EDTA, 1% NP-40, 0.1% SDS, 1 mM phenylmethylsulfonyl fluoride, and cOmplete mini protease inhibitor (Roche), the proteins were separated by SDS-PAGE, transferred to a nitrocellulose membrane, probed with appropriate target-specific primary Abs (1:1,000), decorated by a peroxidase (HRP)-conjugated secondary Abs (1:5,000), and detected with enhanced chemiluminescent West Femto maximum sensitivity substrate (Pierce), using a GBOX-Chemi-XRQ-E system (Syngene, Frederick, MD, USA). Densitometric quantification of immunoblots was performed using the ImageJ software from NIH (https://imagej.nih.gov/ij/).

### Quantitative real-time PCR

Total RNA was isolated using RNA Blue (TopBio, Czech Republic) and subsequently treated with DNAse before it was reverse transcribed to cDNA using a High-Capacity reverse transcription kit (Applied Biosystems). Triplicate quantitative real-time PCR (qRT-PCR) reactions (10 µL) were run in HOT FIREPol EvaGreen qPCR Supermix (Solis Biodyne) with 0.8 µM primers (Table S1) on a Bio-Rad CFX384 Real-Time PCR System. The 2^ΔΔCt^ values were determined and the mRNA levels were normalized to that of the human β2-microglobulin and β-actin transcript.

### Analysis of quenchable iron pool

The quenchable iron pool (QIP) was determined as described previously (46). Monocytes exposed for 2 days to 4 ng/mL of CyaA, or the CyaA-AC^−^ toxoid were washed twice with pre-warmed Hanks' balanced salt solution (HBSS) (w/o ions), transferred from six-well plates into a 96-well round bottom plate, washed again with HBSS (w/o ions) and resuspended in 100 µL of 0.1 µM Calcein-AM in HBSS without Mg^2+^ and Ca^2+^ ions. The cells were incubated at 37°C, 5% CO_2_ for 8 min, washed three times with 200 µL pre-warmed HBSS (w/o ions), and split into two 100 µL aliquots. One was left untreated and the other 100 µL of Calcein-AM treated cells was mixed with 100 µL of 5 µM FeCl_2_, 10 µM 8-hydroxyquinoline solution in HBSS (w/o ions), and incubated at 37°C for 8 min. Cells were washed twice with ice-cold HBSS (with Mg^2+^ and Ca^2+^ ions) and fluorescence of calcein was measured upon excitation at 488 nm using a FITC emission filter (530/20 nm) on an LSR II flow cytometer (BD Biosciences). QIP was calculated as described previously (46) as QIP = MFI(untreated cells) – MFI(FeHQ-treated cells) (MFI, median fluorescence intensity) and the percentage of QIP = 100 × QIP_sample_/QIP_control_.

### Statistics

Statistical analysis was performed using the GraphPad embedded algorithm by paired *t* test throughout the manuscript.

## RESULTS

### Adenylate cyclase toxin through cAMP/PKA-mediated signaling blocks M-CSF-induced upregulation of iron acquisition receptors CD71 and CD163 on monocytes

**Fig 1:**
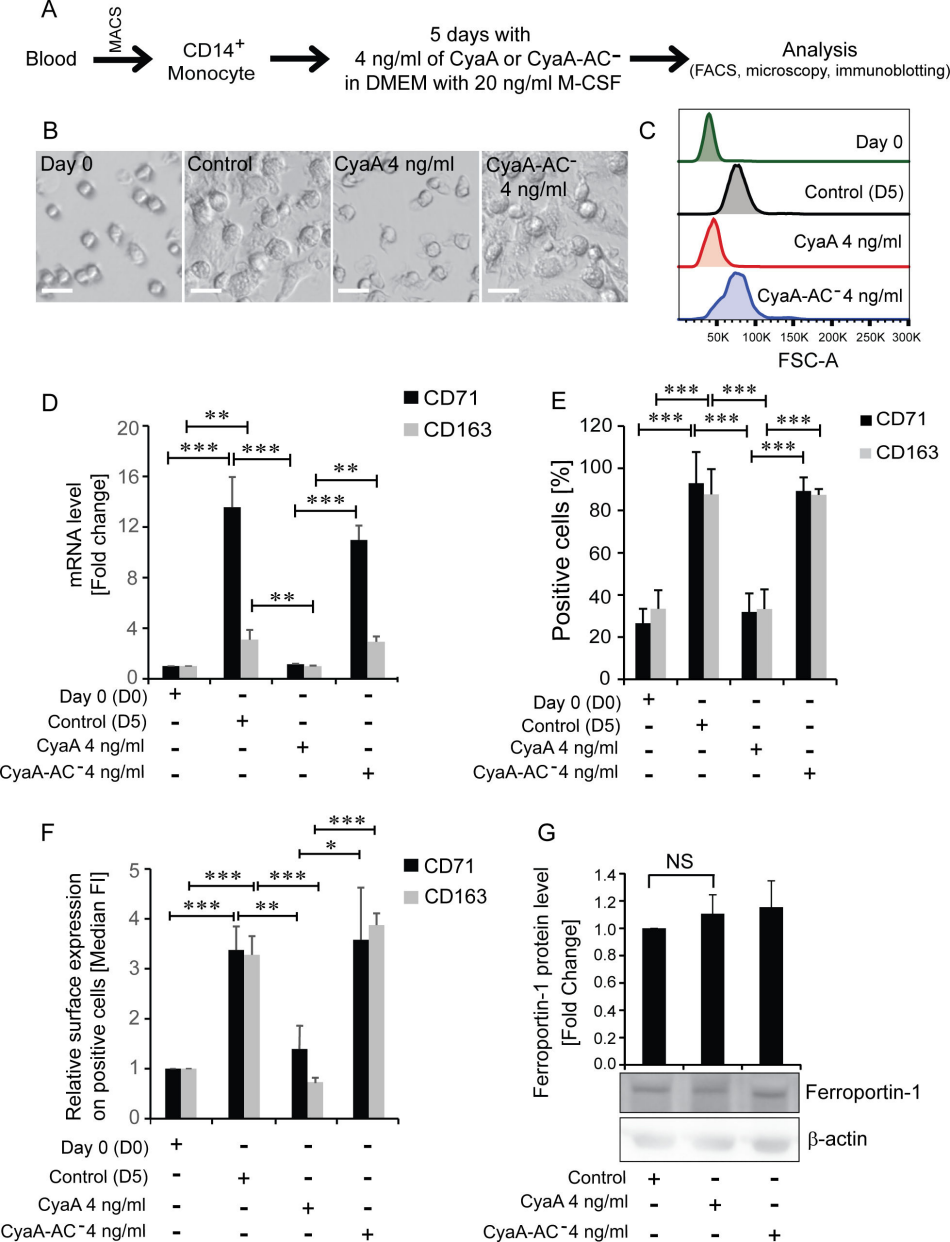
CyaA-exposed CD14^+^ monocytes are unable to acquire the large macrophage cell features and lose CD71 and CD163 expression. (**A**) Scheme of the experiment. CD14^+^ monocytes were purified from PBMCs using CD14 MicroBeads on MACS, cultured with 20 ng/mL M-CSF with or without 4 ng/mL CyaA for 5 days, and analyzed by flow cytometry, microscopy, and immunoblotting. (**B**) CyaA (22.5 pM) blocks M-CSF-driven monocyte cell enlargement. Over 5 days of culture in the presence of 20 ng/mL of M-CSF, the mock-treated control cells and the 22.5 pM CyaA-AC^−^-treated cells acquired features of large adherent macrophage cells. In contrast, monocytes exposed to 22.5 pM (4 ng/mL) CyaA resembled the undifferentiated cells from day 0 and remained small and partially adherent. One brightfield image representative of at least 12 images taken per condition is shown; scale bar 20 µm, magnification 20× with 2× zoom. (**C**) CyaA blocks cell enlargement, and cell size after 5 days of M-CSF stimulation in the presence or absence of enzymatically active 4 ng/mL (22.5 pM) CyaA was determined by fluorescence-activated cell sorting (FACS) analysis as forward scatter and compared to the size of CD14^+^ monocytes on day 0. One representative histogram out of at least six independent biological replicates (*n* ≥ 6) is shown. (**D**) *CD71* and *CD163* transcript levels quantified by RT-qPCR in 22.5 pM CyaA-treated CD14^+^ monocytes of three different donors were normalized to the level of β_2_-microglobulin and β-actin mRNA and plotted as fold increase over mock controls. Untreated day 0 cells were used for basal transcript level determination for fold change calculations. Means ± SD values are shown (*n* = 3). (**E**) Percentage and (**F**) median fluorescence intensity of CD71 and CD163 positive cells as determined by flow cytometry analysis of surface expression of CD71 and CD163 on CD14^+^ monocytes cultured for 5 days with 20 ng/mL of M-CSF and either with 4 ng/mL CyaA, or with 4 ng/mL of the CyaA-AC^−^ toxoid. Untreated day 0 cells were used for basal level determination. No CyaA-derived protein was added to mock-treated control cells (*n* > 6). (**G**) Western blot detection of Ferroportin-1 protein after 5 days of CyaA toxin or mutant toxoid treatment, β-actin is acting as a loading control (*n* = 3). ***P < 0.0005, **P < 0.005, *P < 0.05. Mock-treated cells exposed to buffer only and cultured for 5 days with 20 ng/mL of M-CSF were used as controls.

We have previously reported that cAMP-dependent protein kinase A (PKA) signaling, elicited by as little as 22.5 pM CyaA toxin, completely blocks M-CSF-driven differentiation of primary human blood monocytes into M2 type of macrophages (13). Since iron acquisition is crucial for cell size expansion and organelle formation during monocyte-to-macrophage transition (25–28), we examined if the cAMP signaling elicited by CyaA altered the surface levels of cellular endocytic receptors involved in iron delivery into differentiating monocytes. Toward this aim, primary human CD14^+^CD16^−^ blood monocytic cells were separated on magnetic beads to >90% purity (Fig. S1) and the cells were exposed to 4 ng/mL (22.5 pM) CyaA for 5 days in DMEM with 20 ng/mL of M-CSF (Fig. 1A). This low toxin concentration was used because preliminary experiments indicated that it was sufficient for blocking of upregulation of the CD71 and CD163 iron uptake receptors during the process of M-CSF-driven monocyte differentiation (Fig. S2). In line with previously reported results (13), exposure to 4 ng/mL CyaA toxin in the presence of 20 ng/mL of M-CSF blocked monocyte transition into the large, differentiated macrophage cells, whereas monocytes incubated with the enzymatically inactive CyaA-AC^−^ toxoid that does not elevate cellular cAMP, differentiated into macrophages like the mock-treated control cells (Fig. 1B). The CyaA-exposed monocytes retained the same appearance as the initial monocytes (day 0), their size did not change after 5 days of incubation in presence of the toxin (Fig. 1C) and expressed significantly lower levels of mRNA for the CD71 and CD163 proteins compared to mock-treated control cells differentiated for 5 days with 20 ng/mL of M-CSF (Fig. 1D). In line with that, the percentage of CyaA-treated cells that expressed the CD71 and CD163 receptors on cell surface remained as low as in the initial suspension of undifferentiated monocytes on day 0. In contrast, almost 100% of cells that differentiated in the presence of CyaA-AC^−^ toxoid expressed the CD71 and CD163 receptors after 5 days of incubation with M-CSF, as the mock-treated control cells (Fig. 1E). Moreover, the CyaA toxin-treated cells expressed significantly lower amounts of the iron acquisition receptors CD71 and CD163 also on a per cell basis, compared to the differentiating mock-treated control cells, or cells cultured in the presence of the CyaA-AC^−^ toxoid (Fig. 1F). Therefore, we analyzed if CyaA action also impacted on the levels of the iron exporter Ferroportin-1 (Slc40a1, FPN) that is involved in the buffering of the intracellular iron pool. However, no significant change in FPN protein expression was observed on CD14^+^ monocytes exposed to 4 ng/mL of CyaA for 5 days, and the FPN level was similar to that of cells exposed to the enzymatically inactive CyaA-AC^−^ toxoid or in mock-treated control cells (Fig. 1F). It can thus be concluded that CyaA action only affected the iron acquisition system and export of iron from CyaA-exposed CD14^+^ monocytes was likely not altered.

**Fig 2:**
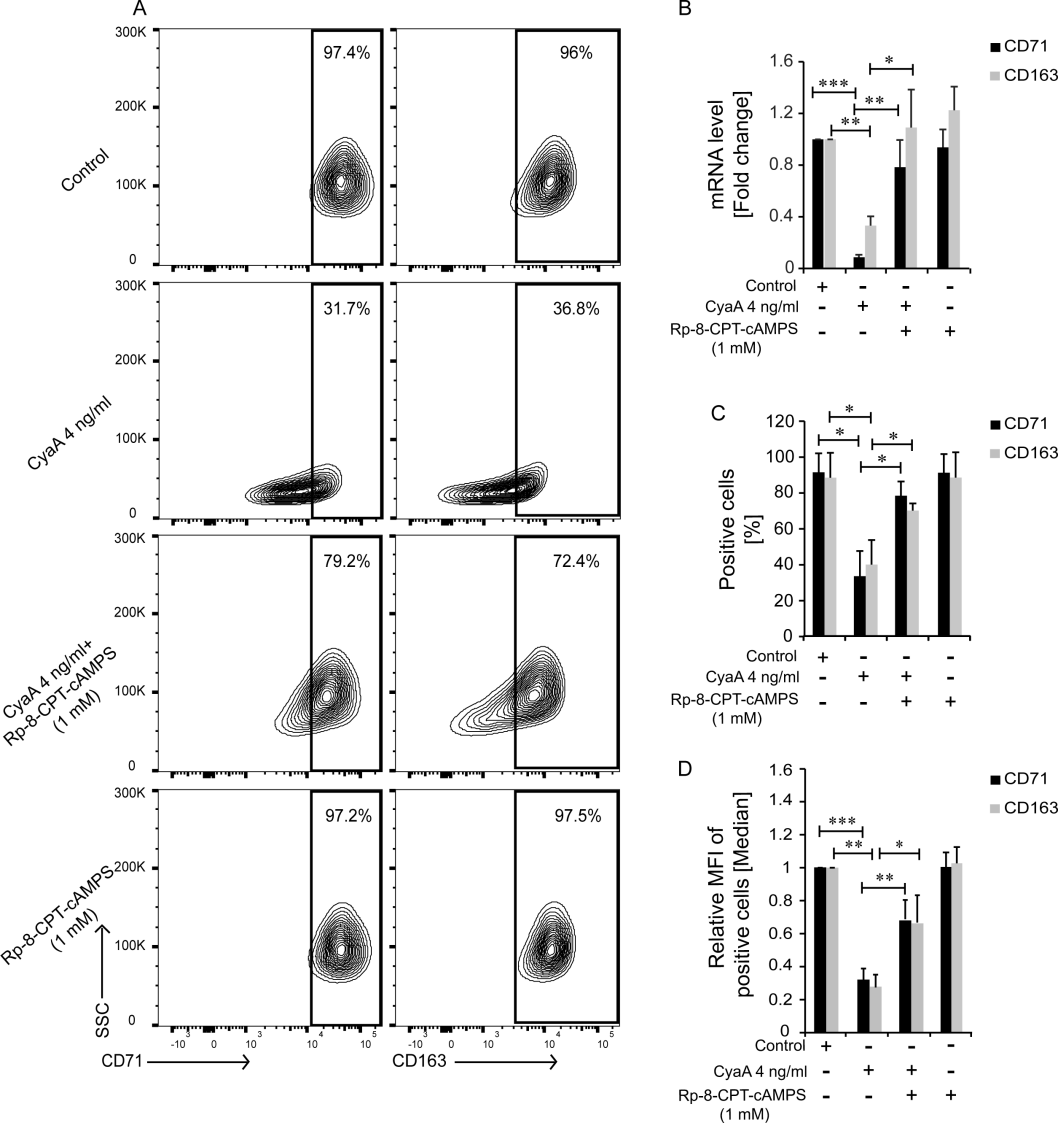
CyaA-produced cAMP signaling through PKA downregulates the M-CSF-driven CD71 and CD163 expression on monocytes. (**A**) CD71 and CD163 surface levels on CD14^+^ monocytes cultured with 20 ng/mL M-CSF for 5 days in the presence of 4 ng/mL of CyaA with or without PKA inhibitor Rp-8-CPT-cAMPS (1 mM). Mock-treated control cells or CyaA-AC^−^-treated cells served as control. Representative contour plots of data from one of three independent experiments performed on cells from different donors are shown. At least 10,000 cells per condition were analyzed and the percentage of cells expressing CD71 and CD163 is indicated in the boxes. (**B**) *CD71* and *CD163* transcript levels in CD14^+^ monocytes exposed to 4 ng/mL of CyaA-AC^−^ or to 4 ng/mL CyaA, with or without 1 mM Rp-8CPT-cAMPS (*n* = 3), transcript level was normalized to β_2_-microglobulin and β-actin mRNA. (**C**) Percentage of CD71 and CD163 expressing cells is shown, following 5 days of exposure to 4 ng/mL of CyaA in the presence or absence of 1 mM of Rp-8-CPT-cAMPS or cells exposed to 1 mM Rp-8-CPT-cAMPS (*n* = 3). (**D**) Flow cytometry analysis of cell surface expression of relative levels of cell surface expression of CD71 and CD163 on positive cells is shown upon 5 days of exposure to CyaA with or without 1 mM Rp-8-CPT-cAMPS or to 1 mM Rp-8-CPT-cAMPS. PKA inhibitor Rp-8-CPT-cAMPS (1 mM) partially alleviated the CyaA-mediated block of M-CSF-induced upregulation of CD71 and CD163; the MFI values shown represent means ± standard deviations (SD). *n* = 3; ***P < 0.0005, **P < 0.005, *P < 0.05.

Previously, we found that action of the very low CyaA amounts (22.5 pM) blocked monocyte differentiation primarily through cAMP-mediated activation of the PKA. Since PKA gene silencing and transfectant sorting would have interfered with monocyte differentiation, we used the highly PKA-specific inhibitor Rp-8-CPT-cAMPS (47) to examine if PKA activation by CyaA-produced cAMP accounted also for the downregulation of expression of the CD71 and CD163 receptors. Pretreatment of CD14^+^ monocytes with Rp-8-CPT-cAMPS (1 mM) alone had no effects whatsoever on their M-CSF-driven differentiation, compared to mock-treated control cells (Fig. 2A). However, pretreatment with 1 mM Rp-8-CPT-cAMPS alleviated to large extent the CyaA-imposed inhibition of CD71 and CD163 expression, restoring the levels of *CD71* and *CD163* mRNA in the CyaA-exposed cells and thus allowing exposure of the receptors on the surface of M-CSF-differentiated monocytes (Fig. 2B). A significantly higher fraction of CyaA-exposed cells treated with 1 mM Rp-8-CPT-cAMPS expressed CD71 and CD163 on cell surface and the median fluorescence intensities of detected CD71 and CD163 amounts on such cells were also about twice higher than in the absence of the PKA inhibitor (Fig. 2C and D). It can, hence, be concluded that PKA activation by CyaA-produced cAMP accounted for the block of M-CSF-induced CD71 and CD163 expression on CD14^+^ monocytes that failed to undergo the M-CSF-driven differentiation.

**Fig 3:**
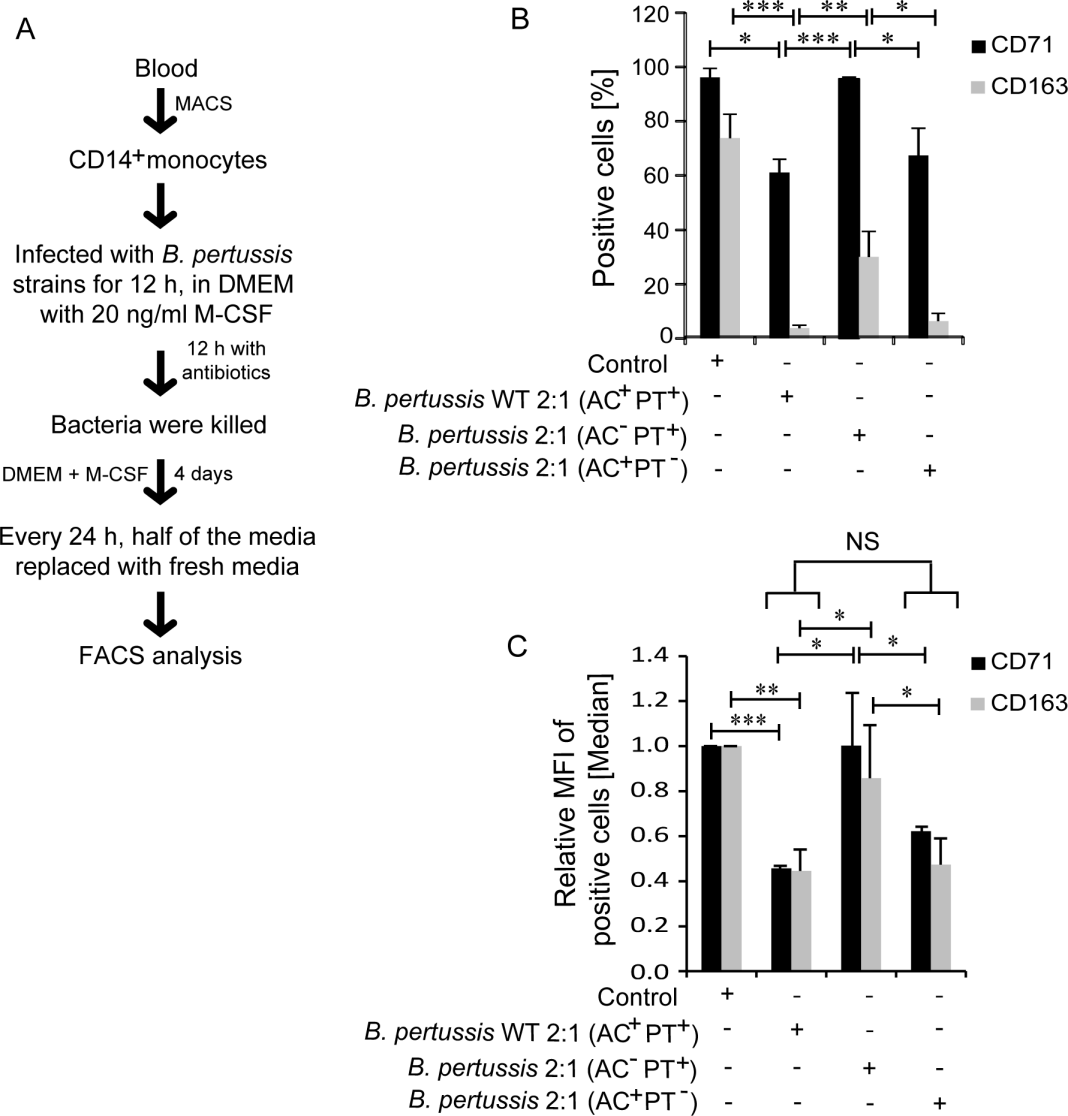
*B. pertussis* CyaA alone downregulates M-CSF-induced CD71 and CD163 expression on CD14^+^ monocytes. (**A**) Scheme of the experiment. CD14^+^ monocytes were infected with indicated *B. pertussis* CIP 81.32 Tohama I-derived strains at MOI 2:1 for 12 h before the bacteria were killed with antibiotics (50 µg/mL polymyxin B plus 50 µg/mL kanamycin) for additional 12 h. Half of the medium containing antibiotic cocktail (Sigma) was replaced every 24 h. (**B**) The percentage of CD71 and CD163-expressing cells was assessed by flow cytometry. (**C**) The median fluorescence intensity of CD71 and CD163 on the positive cells from three donors is shown as means ± SD values (*n* = 3). ***P < 0.0005, **P < 0.005, *P < 0105; NS, not significant.

To explore whether additional *B. pertussis* virulence factors or bacteria-released TLR ligands also impacted CD71 and CD163 expression, we infected the M-CSF-stimulated CD14^+^ monocytes with *B. pertussis* or with its isogenic mutants producing individually, or in combination, the enzymatically inactive toxoids of CyaA or PT, respectively (13). Bacteria were co-cultured with cells at a very low multiplicity of infection (MOI=2:1) for 12 h before the bacteria were killed by antibiotics (50 µg/mL polymyxin B and 50 µg/mL of kanamycin) to interrupt toxin production. After an additional 12 h, the monocytes were washed and cultured for 4 days in the presence of 20 ng/mL of M-CSF prior to cytometric analysis (Fig. 3A). As shown in Fig. 3B, compared to mock-treated cells (control), or the monocytes infected by the *B. pertussis* (AC^−^PT^+^) mutant producing active PT and the enzymatically inactive CyaA-AC^−^ toxoid (Table 1), which differentiated and expressed higher levels of CD71 and CD163 (Fig. 3B and C), monocyte infection with the wild-type *B. pertussis* strain producing both toxins (AC^+^PT^+^) provoked a significant decrease in the number of cells that still expressed some CD71 and CD163 receptor on cell surface. Moreover, the remaining positive cells expressed significantly reduced amounts of the respective receptors (Fig. 3C). Moreover, infection by the *B. pertussis* (AC^+^PT^−^) mutant that produced enzymatically inactive PT toxoid but an active CyaA, reduced CD71 and CD163 expression as much as infection by the wild-type bacteria. Hence, the sole cAMP-elevating activity of the CyaA toxin strongly downregulated the M-CSF-triggered surface expression of the iron acquisition receptors CD71 and CD163 in M-CSF-differentiated monocytes also in the context of the infection by live *B. pertussis* bacteria at a very low MOI of 2:1. Under such conditions the capacity of the pertussis toxin to upregulate the cellular cAMP levels through its ability to indirectly deregulate the endogenous cellular adenylyl cyclase activity was insufficient for suppression of CD71 and CD163 expression. It can, hence, be concluded that the action of the *B. pertussis*-produced CyaA was alone sufficient to block the M-CSF-driven expression of iron acquisition receptors in differentiating CD14^+^ monocytes by CyaA-activated PKA signaling.

**TABLE 1:** Description of bacterial strains used in this study

Strain	Genotype and/or phenotype	Source or reference
*B. pertussis* (AC^+^PT^+^)	Tohama I wild-type strain	Institute Pasteur collection, CIP 81.32
*B. pertussis* (AC^−^PT^+^)	ATP binding site of the AC enzyme of CyaA was destroyed by the insertion of a GlySer dipeptide between the residues 188 and 189, yielding a *B. pertussis* mutant producing an enzymatically inactive CyaA-AC^−^ toxoid unable to elevate cAMP	44
*B. pertussis* (AC^+^PT^−^)	S1 subunit of PT with the R9K and E129G substitutions ablating the ADP ribosylation activity, yielding a *B. pertussis* mutant producing an enzymatically inactive PT toxoid	48
*E. coli* (XL1-Blue)	Carries mutant alleles of *recA1, endA1, gyrA96, thi-1, hsdR17, supE44, relA1, lac*	Stratagene, La Jolla, CA

### CyaA decreases HO-1 level in CD14^+^ monocytes and upregulation of HO-1 does not relieve the CyaA-imposed inhibition of monocyte-to-macrophage transition

**Fig 4:**
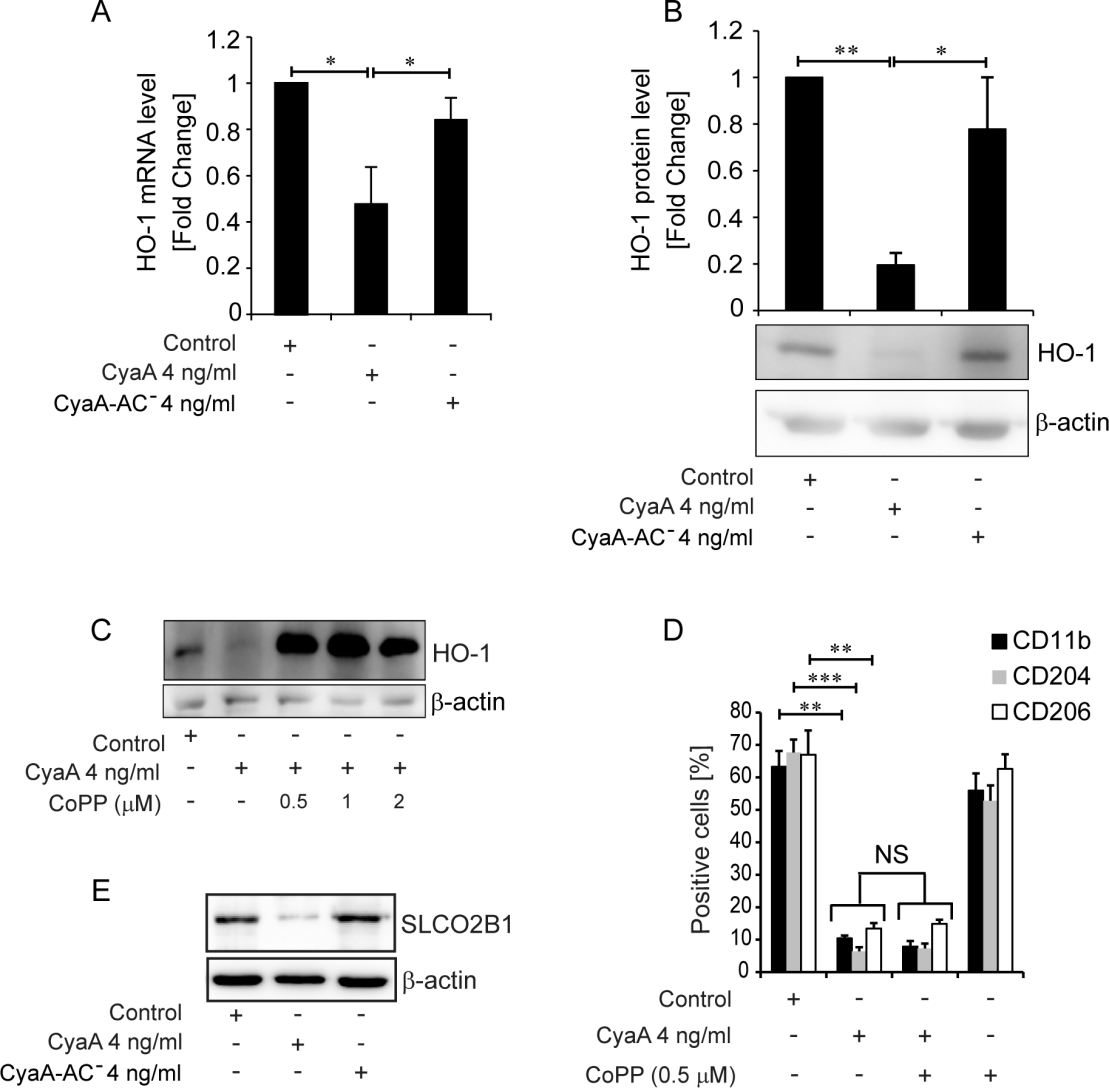
CyaA downregulates HO-1 expression and HO-1 induction does not relieve the inhibition of monocyte differentiation imposed by CyaA. (**A**) HO-1 transcript levels in CD14^+^ monocytes cultured for 5 days with 4 ng/mL of CyaA. HO-1 expression was normalized to β_2_-microglobulin and β-actin transcript level and the mean ± SD of values from three independent determinations on cells of different donors are shown. (**B**) HO-1 protein levels are determined in cell lysates by densitometry of immunoblot signals, using β-actin detection for normalization of the signal. (**C**) Immunoblot detection of HO-1 induction by CoPP, using β-actin detection as a loading control. (**D**) HO-1 induction by 0.5 µM CoPP does not relieve the CyaA-imposed block of monocyte differentiation, CD14^+^ monocytes were cultured with 4 ng/mL of CyaA for 5 days in the presence or absence of 0.5 µM CoPP, and cell surface expression of the macrophage markers CD11b, CD204, and CD206 was analyzed by flow cytometry. (**E**) The alternative heme importer protein SLCO2B1 levels were probed by immunoblot of cell lysates using specific antibody and β-actin levels serving as loading control. ***P < 0.0005, **P < 0.005, *P < 0.05; NS, not significant.

The liberation of iron from internalized heme by the HO-1 enzyme represents an important step in iron acquisition. We thus examined if CyaA-elicited cAMP signaling also regulated HO-1 levels. Indeed, RT-qPCR analysis revealed an about twofold decrease of *Hmox1* mRNA level in monocytes cultured with 4 ng/mL CyaA, as compared to mock-treated or CyaA-AC^−^-exposed cells (Fig. 4A). The CyaA-treated cells then produced about ~80% less of the HO-1 protein, as determined by densitometric analysis of immunoblots (Fig. 4B), suggesting that low HO-1 levels in CyaA-treated cells potentiate the block of iron acquisition by reduced activity of the machinery that liberates iron from the residual heme acquired via CD163.

We thus examined if HO-1 expression can still be induced in toxin-treated monocytes and could promote their differentiation into macrophages. In fact, as low as 0.5 µM concentration of the HO-1 inducer CoPP could completely override the inhibition of HO-1 protein production elicited by CyaA action (Fig. 4C). Nevertheless, despite producing large amounts of HO-1 upon induction by 0.5 µM CoPP, the CyaA toxin-exposed monocytes were still unable to differentiate into macrophage cells and failed to upregulate the expression of the M-CSF-driven M2 macrophage maturation markers (49) CD11b, CD204, and CD206 (Fig. 4D). We thus examined if CyaA action affected also the levels of the newly discovered alternative heme importer SLCO2B1 (50). As shown in Fig. 4E, importantly reduced amounts of the SLCO2B1 protein were detected in cells cultured in the presence of 4 ng/mL CyaA, compared to mock control or toxoid-exposed cells. Hence, CyaA action downregulated both known heme importers, CD163 and SLCO2B1.

### CyaA-exposed cells contain reduced intracellular iron levels and exogenous iron supply does not alleviate the CyaA-triggered differentiation block

**Fig 5:**
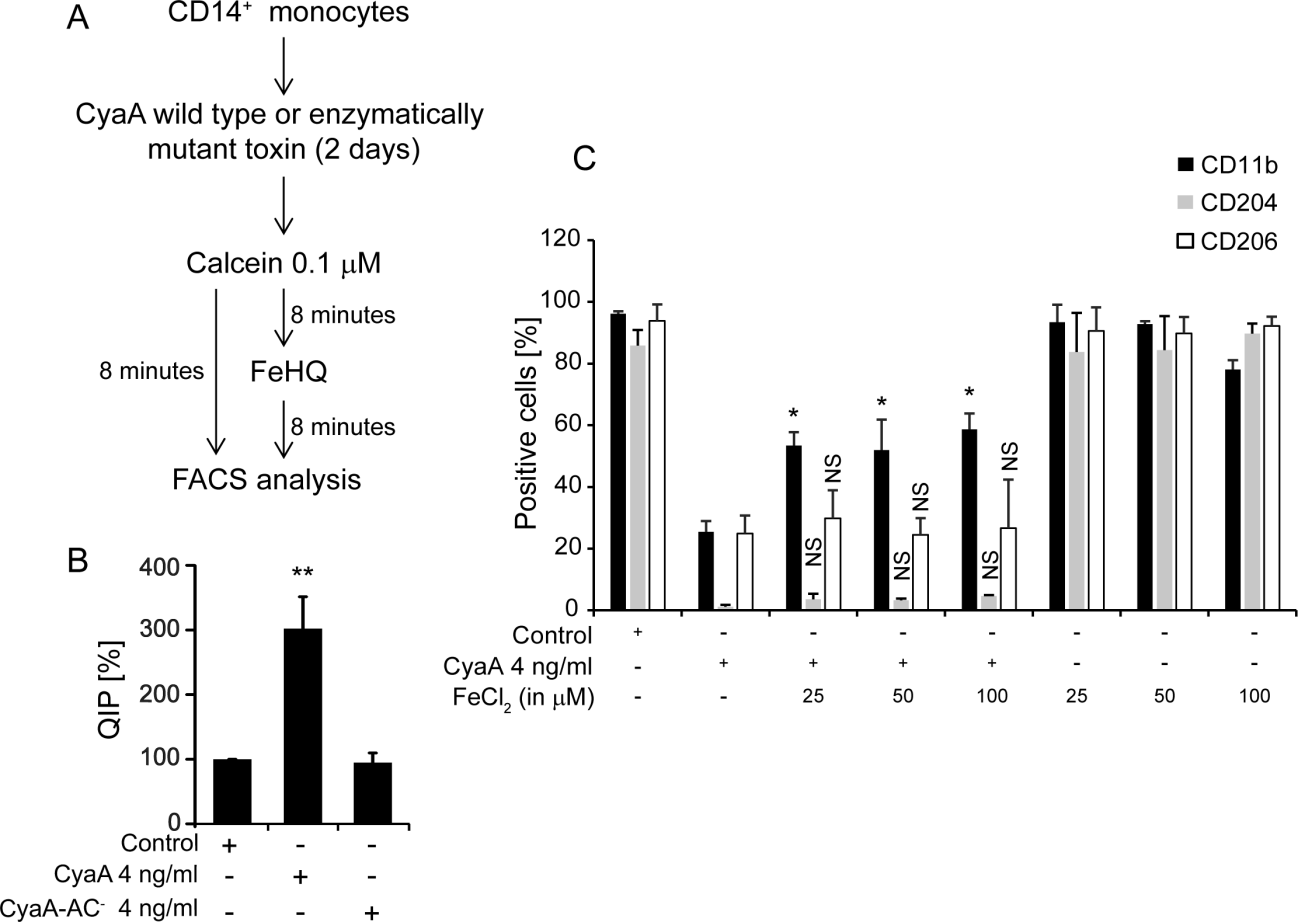
CyaA triggers depletion of intracellular iron pool and excess extracellular iron does not relieve the CyaA-imposed block of M-CSF-driven differentiation of monocytes. (**A**) CD14^+^ monocytes cultured for 2 days with 4 ng/mL of CyaA or mutant CyaA-AC^−^ toxin in the presence of human M-CSF (20 ng/mL) were stained with Calcein-AM (0.1 µM) for 8 min. Cell suspensions were split in two and one aliquot of each was supplemented with FeHQ complexes [FeCl_2_ (F.C. 5 µM) and 8-HQ (F.C. 10 µM)] for an additional 8 min at 37°C before the fluorescence of the internalized calcein probe was measured by flow cytometry, using excitation at 488 nm and detection at 530 nm, respectively. (**B**) The calcein probe fluorescence intensity before and after FeHQ was utilized to calculate the QIP = MFI(untreated cells) – MFI(FeHQ-treated cells) and the % QIP = 100 × QIP_sample_/QIP_control_. *n* = 4, *P < 0.05, **P < 0.005. (**C**) CD14^+^ monocytes were exposed to 4 ng/mL of CyaA in the presence or absence of indicated amounts of FeCl_2_ for 5 days with 20 ng/mL M-CSF in DMEM. The percentage of cells expressing macrophage markers CD11b, CD204, and CD206 was determined by flow cytometry *n* = 3, *P < 0.05.

Accessible intracellular iron is critical for the proper functioning of any mammalian cell. As the CyaA-elicited cAMP signaling reduced the expression of iron acquisition receptors on CD14^+^ monocytes, we assessed if CyaA action resulted in the depletion of the intracellular Fe^2+^ pool in monocytes exposed to the M-CSF differentiation signal. This was assessed after 2 days of exposure of the cells to the toxin, at which time the difference in cell size between the differentiating control monocytes and of the non-differentiating cells is not yet too important, so that it does not bias the readout of the assay based on quenching of fluorescence of the internalized calcein probe by intracellular free Fe^2+^ ions. The CD14^+^ monocytes were exposed to 4 ng/mL of CyaA or of the enzymatically CyaA-AC^−^ toxoid for 2 days and the QIP was assessed by calcein-AM staining (46). Since calcein fluorescence is quenched in the iron-bound state, addition of 5 µM FeCl_2_ with 10 µM 8-hydroxyquinoline (HQ) mixture to cells normally leads to swift delivery of the formed FeHQ complexes into cell cytoplasm, which increases cytosolic Fe^2+^ concentration and causes quenching of calcein fluorescence (46, 51). Moreover, at low cytoplasmic iron levels, the cells have an increased capacity to take up the exogenous FeHQ complexes. Indeed, as shown in Fig. 5B, the monocytes cultured for 2 days with 4 ng/mL of CyaA were iron-starved and took up the added FeHQ rapidly, exhibiting an almost threefold increase of calcein fluorescence quenching (QIP). In contrast, the QIP values of the mock or CyaA-AC^−^-treated cells did not change upon FeHQ addition. Hence, only the CyaA-exposed monocytes were iron-starved under the used experimental conditions of culture in DMEM medium containing 20 ng/mL of M-CSF. Indeed, the iron starvation and the block of monocyte differentiation was not overcome upon increase in free iron ion concentration in the media (25 to 100 µM FeCl_2_) despite the presence of transferrin supplied by the 10% FCS used, further pointing to inhibition of iron import by suppression of CD71 expression (Fig. 5C).

### Inhibition of HDAC activity alleviates the CyaA-elicited inhibition of CD71 and CD163 expression

**Fig 6:**
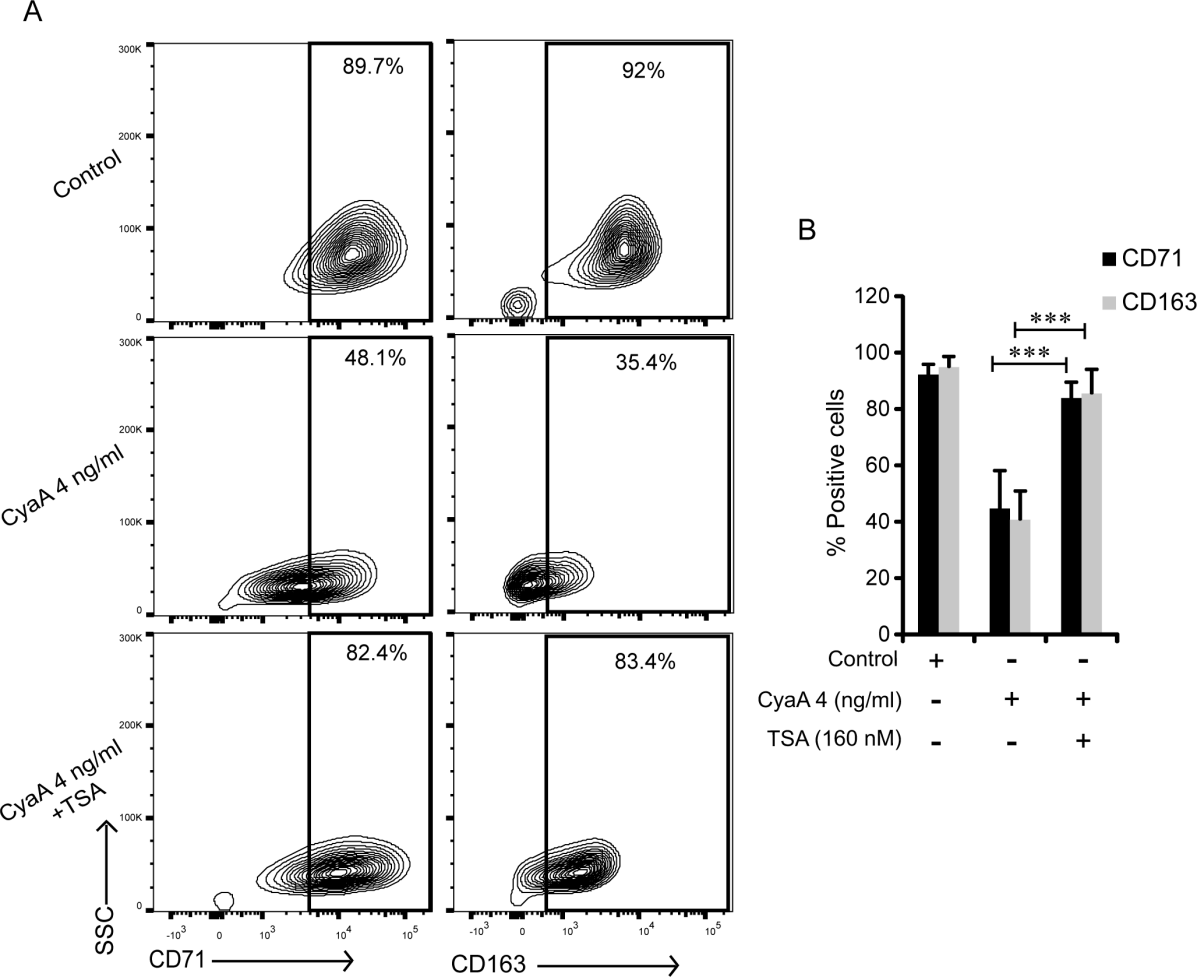
HDACs inhibitor TSA alleviates CyaA-mediated decrease of CD71 and CD163 expression on CD14^+^ monocytes. CD14^+^ monocytes were cultured for 5 days in media containing 20 ng/mL M-CSF and 4 ng/mL of CyaA, with or without the HDAC inhibitor TSA at 160 nM concentration. Cells were analyzed by flow cytometry and (**A**) contour plots from one experiment representative of four independent biological replicates are shown (*n* = 4). At least 10,000 cells per condition were analyzed and the percentage of cells expressing CD71 and CD163 is indicated in the boxes. (**B**) Upon 5 days of exposure of CD14^+^ monocytes to 4 ng/mL of CyaA in the presence or absence of 160 nM of TSA, the means of percentage of CD71^+^ and CD163^+^ cells were calculated using values from four independent biological replicates performed on monocytes of four different donors. ***P < 0.0005.

cAMP signaling through PKA was previously reported to regulate the activity of a wide range of histone deacetylases (HDACs) that are involved in the epigenetic regulation of gene expression (52–54). Therefore, we used the class I/II HDACs inhibitor trichostatin A (TSA) to assess the involvement of HDACs in CyaA-PKA-mediated downregulation of CD71 and CD163 expression on CD14^+^ monocytes. As shown in Fig. 6, the presence of TSA (160 nM) over 5 days of exposure of monocytes to 4 ng/mL of CyaA largely alleviated the CyaA/cAMP signaling-mediated block of M-CSF-induced expression of both receptors, yielding a markedly increased number of cells expressing CD71 and CD163 (Fig. 6A and B). Hence, the CyaA/cAMP signaling through HDACs causes silencing of the M-CSF-induced expression of the iron acquisition receptors CD71 and CD163.

## DISCUSSION

To proliferate on host airway mucosa, *B. pertussis* needs to evade the bactericidal action of patrolling and mucosa-infiltrating phagocytes. We found that cAMP signaling elicited by as little as 4 ng/mL (22.5 pM) CyaA toxin blocks the M-CSF-induced upregulation of expression of iron acquisition receptors CD163 and CD71 required for meeting of the increased iron supply requirements of enlarging monocytes in the course of differentiation into the macrophage cells. By limiting the increase in intracellular iron (Fe^2+^) availability (c.f. Fig. 5), this effect of CyaA/cAMP signaling would then participate in the toxin-imposed inhibition of monocyte to macrophage differentiation.

The threefold enlargement of the bactericidal macrophage cells, compared to the precursor cell, depends on the upregulation of cellular metabolism of the differentiating monocytes, which includes increased cellular respiration and DNA synthesis. All these processes largely depend on the level of available intracellular iron and are not efficiently operating in low iron conditions. The transition of mucosa-infiltrating inflammatory monocytes into the much larger macrophage cells thus requires increased iron acquisition capacity in support of cell growth and for the proliferation of mitochondria and other organelles. Therefore, limiting of the iron acquisition capacity of monocytes, as one of the many consequences of CyaA-elicited cAMP signaling, likely contributes importantly to the block of differentiation of CyaA-exposed monocytes to macrophages. It would go well with a generalized transcriptional silencing that appears to be due to epigenetic changes and heterochromatin accumulation in toxin-treated cells that we have recently observed ( J. N. Ahmad, M. Modrak, M. Fajfrova, O. Benada, and P. Sebo, submitted for publication). In line with this, we report here that inhibition of histone deacetylases by TSA largely alleviated the CyaA-elicited block of M-CSF-triggered upregulation of the CD71 and CD163 iron acquisition receptors (c.f. Fig. 6). This further points to an epigenetic mechanism of silencing of the expression of genes required for the enhanced iron acquisition needs of differentiating monocytes.

We have previously shown that, through cAMP signaling and PKA activation, already very low CyaA amounts triggered the de-differentiation of primary human alveolar macrophages back into monocyte-like cells (13). The use of bacterial mutants producing enzymatically inactive PT or CyaA toxoids now revealed that CyaA alone was the key virulence factor of *B. pertussis* that efficiently blocked the upregulation of CD71 and CD163 receptors on M-CSF-stimulated monocytes even at the very low MOI of 2 bacteria per one monocytic cell (c.f. Fig. 3). The CyaA-elicited cAMP signaling, hence, blocked the inherent capacity of the cells to respond to intracellular iron shortage by upregulation of expression of iron acquisition receptors (55, 56). Moreover, CyaA-produced cAMP signaling blocked also the subsequent step of iron acquisition by strongly suppressing the expression of the HO-1 enzyme that liberates iron from the heme internalized in complex with the CD163 receptor, or of SLCO2B1 that serves as an alternate heme importer (c.f. Fig. 4). Such combined impact thus provided for a fail-safe block of upregulation of iron acquisition, where upregulation of HO-1 production alone by CoPP did not rescue the differentiation of CyaA-treated monocytes (c.f. Fig. 4D).

The block of transferrin receptor (CD71) upregulation by CyaA further restricted the capacity of monocytes to increase iron acquisition through endocytosis of transferrin-bound iron ions. Indeed, a plausible mechanism behind this block would be the previously described cAMP-triggered downregulation of the phospholipase A2 activity that is required for transferrin receptor recycling (57–59). Hence, the block of upregulated CD71 surface exposure may reflect a combined effect of CyaA-elicited inhibition of CD71 upregulation and cAMP-triggered block of CD71 recycling.

**Fig 7:**
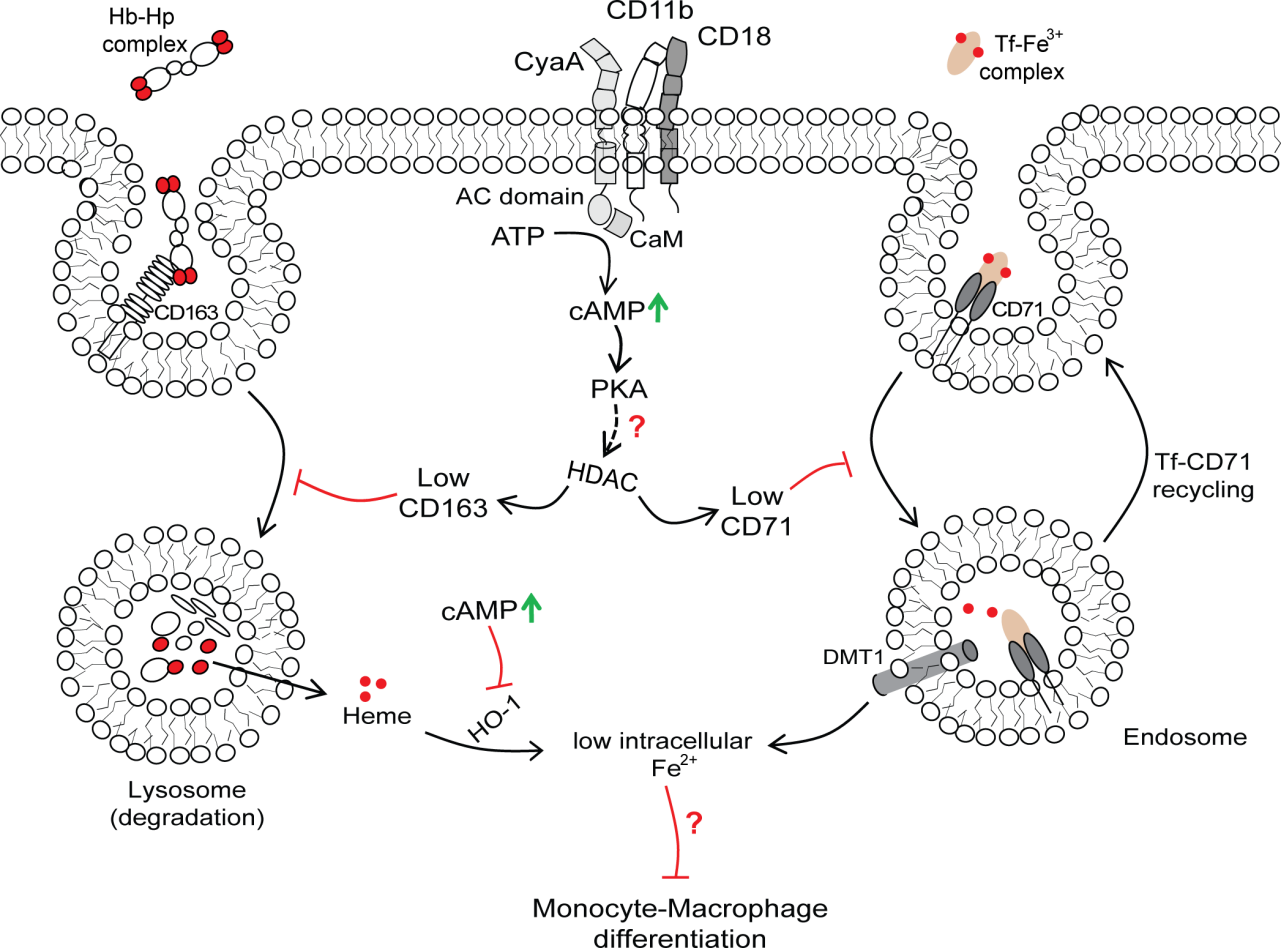
Model of CyaA-mediated block of iron acquisition mechanisms in monocytes. *B. pertussis* CyaA recognizes CD11b/CD18 molecules on CD14^+^ monocytes to translocate its AC enzyme into cell cytosol, where the AC is activated by cytosolic calmodulin (CaM) and catalyzes unregulated conversion of cellular ATP to cAMP, which activates the PKA. CyaA-PKA-cAMP signaling then blocks the nuclear export of HDACs, thereby preventing upregulation of CD71 and CD163 expression in M-CSF-stimulated CD14^+^ monocytes in response to increased iron supply requirements. *B. pertussis* CyaA produced cAMP signaling likely also blocks the Tf-CD71 recycling through inhibition of PLA2 activity (57–59). In addition, cAMP-activated signaling triggers downregulation of the HO-1 protein levels, blocking the liberation of intracellular iron from the imported heme. Altogether, CyaA/cAMP-elicited inhibition of M-CSF-induced CD71 and CD163 upregulation and decreased HO-1 amounts further limits the acquisition of iron needed for monocyte differentiation (24–28). Red lines indicate inhibition whereas black arrows indicate activation.

The high amounts of Hb-Hp complexes in the airway mucosa represent a major iron source for the differentiation of infiltrating inflammatory monocytes into bactericidal macrophage cells (35, 37). In contrast to erythrocytes, the Hb-Hp complexes can easily cross the endothelial fenestrations of capillaries and in the ﬁrst minutes of airway infection the lamina propria is ﬂooded with plasma exudate (37, 60, 61). This passes further through the epithelial basement membrane and transudates between the epithelial cells that are separated at the base, crossing the tight junctions of an intact epithelial lining without compromising its barrier function, entering the airway lumen (33, 38). Moreover, due to eventual extravasation and recruitment of polymorphonuclear leukocytes and other inflammatory cells, the vascular permeability and plasma leakage from the capillaries are further enhanced, which brings the iron-loaded Hb-Hp and transferrin complexes to the surface of the airway mucosa. Indeed, *B. pertussis* infection is associated with the recruitment of large numbers of inflammatory cells, including monocytes, neutrophils, and eosinophils to the infected airway mucosa and into lung parenchyma (62). Therefore, the capacity of CyaA to block the upregulation of the transferrin and Hb-Hp receptors (CD71 and CD163) and of heme importer SLCO2B1 on M-CSF-activated monocytes is likely to play a crucial role in keeping their iron uptake capacity at a level below the needs of the monocyte-to-macrophage differentiation process. Hence, through limiting of iron acquisition capacities of infiltrating monocytes and by blocking their transition to bactericidal macrophages, the *B. pertussis* bacteria would evade a key sentinel line of innate immune barrier on the airway mucosa, as summarized in Fig. 7.

Indeed, CyaA at sub-nanomolar concentrations (e.g., 50 pM) was shown to near-instantly ablate the oxidative burst and opsonophagocytic killing capacities of neutrophils, to trigger apoptosis of macrophages and to block the antigen-presenting capacities of dendritic cells (14–18, 63, 64). The here-described downregulation of iron acquisition receptors adds a novel layer to the amazingly broad array of mechanisms by which CyaA action subverts the bactericidal functions of myeloid phagocytes. It will be important to examine if other bacterial pathogens producing cAMP-elevating toxins, such as *Bacillus anthracis* secreting the edema factor (65), or *Pseudomonas aeruginosa* injecting the ExoY effector into host cells (66), can also suppress monocyte-to-macrophage transition by downregulation of iron acquisition receptors of monocytes as part of their immune evasion strategies.

The here-reported ability of low amounts of CyaA to effectively limit the increase of iron acquisition capacities of CD11b/CD18-expressing myeloid cells opens an intriguing opportunity to explore the potential of CyaA for the treatment of myelomas. There is ample evidence available that iron supply is critical for the rapid proliferation of cancer cells that overexpress CD71 to meet the high iron demand (67). As a result, uptake of transferrin was found to be about four times higher in malignant than healthy non-malignant cells (68). Moreover, a block of CD71 by specific monoclonal antibodies could severely curb cell size expansion and cancer cell division (69, 70). It thus appears plausible to explore whether CyaA at sub-nanomolar and presumably non-immunogenic concentrations (e.g., 50 pM or less) could be used for targeting malignant myeloid cells to limit their iron acquisition and proliferation capacities and for triggering their apoptosis.

## SUPPLEMENTAL MATERIAL

Fig. S1 msphere.00407-24-s0001.tif Gating strategy for CD14^+^ and CD16^−^ monocyte homogeneity assessment.

Fig. S2 msphere.00407-24-s0002.tif CyaA concentration dependence of CD71 and CD163 expression.

Fig. S3 msphere.00407-24-s0003.tif Viability of cells exposed to increasing CyaA concentration.

Table S1 msphere.00407-24-s0004.docx List of primers used for quantification of gene expression by RT-qPCR.

ASM does not own the copyrights to Supplemental Material that may be linked to, or accessed through, an article. The authors have granted ASM a non-exclusive, world-wide license to publish the Supplemental Material files. Please contact the corresponding author directly for reuse.
