# Supplementary figures and images for: cAMP signaling of Bordetella adenylate cyclase toxin blocks M-CSF triggered upregulation of iron acquisition receptors on differentiating CD14+ monocytes
Source: mSphere. 2024 Jul 30;9(8):e00407-24. doi: 10.1128/msphere.00407-24 (PMC11351043; doi:10.1128/msphere.00407-24)

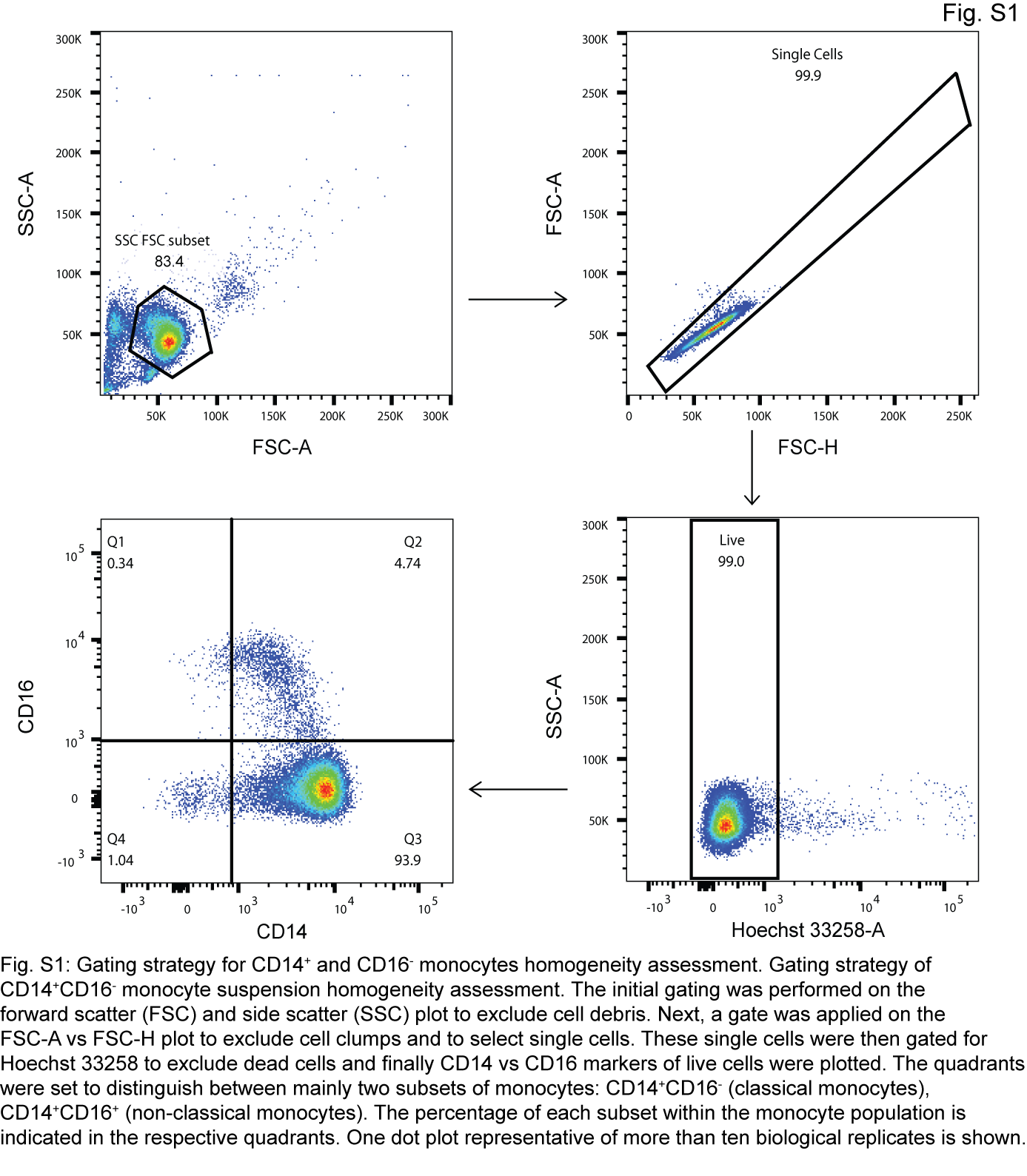

Supplement: Fig. S1 — Gating strategy for CD14+ and CD16− monocyte homogeneity assessment. [file msphere.00407-24-s0001.tif]

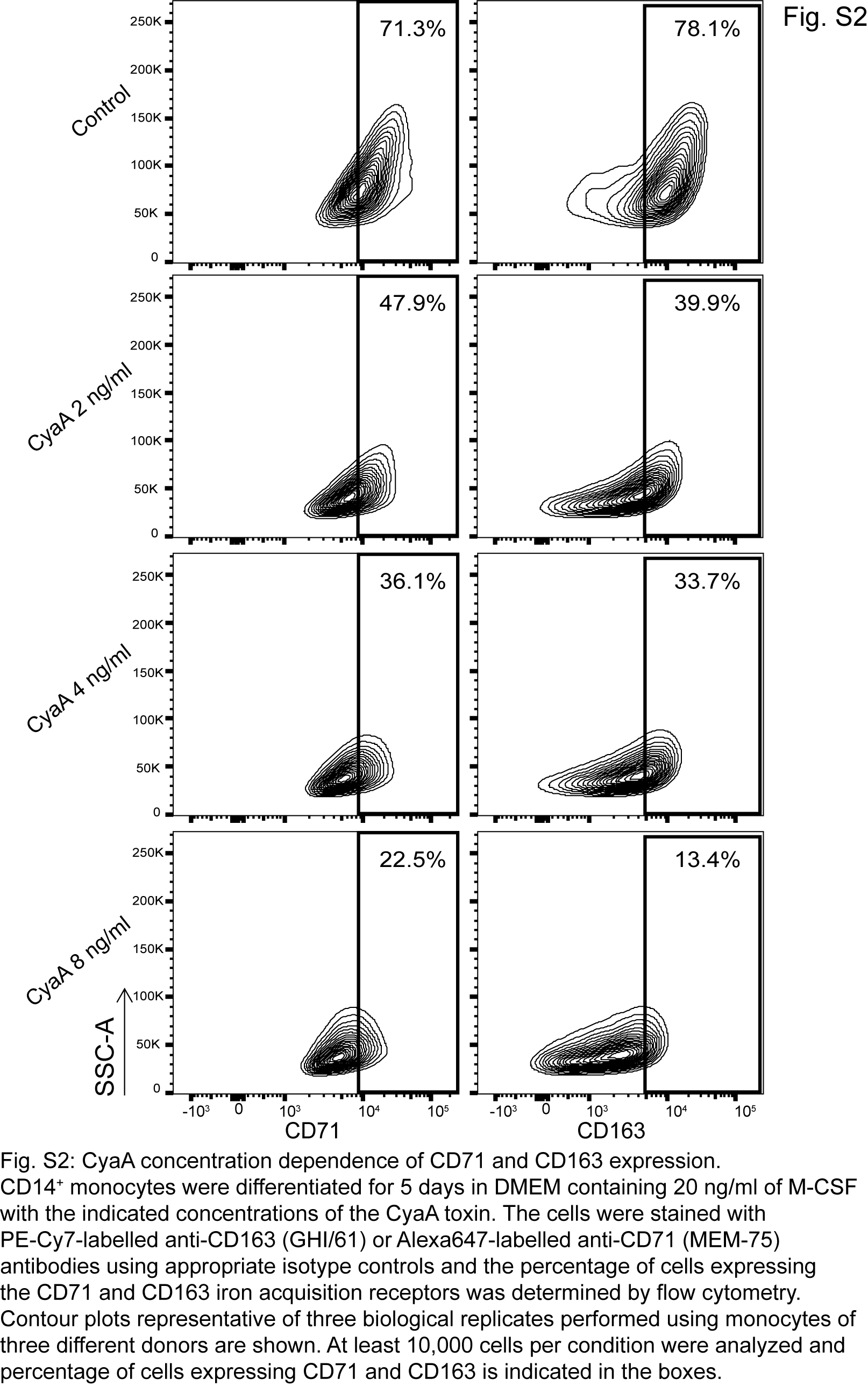

Supplement: Fig. S2 — CyaA concentration dependence of CD71 and CD163 expression. [file msphere.00407-24-s0002.tif]

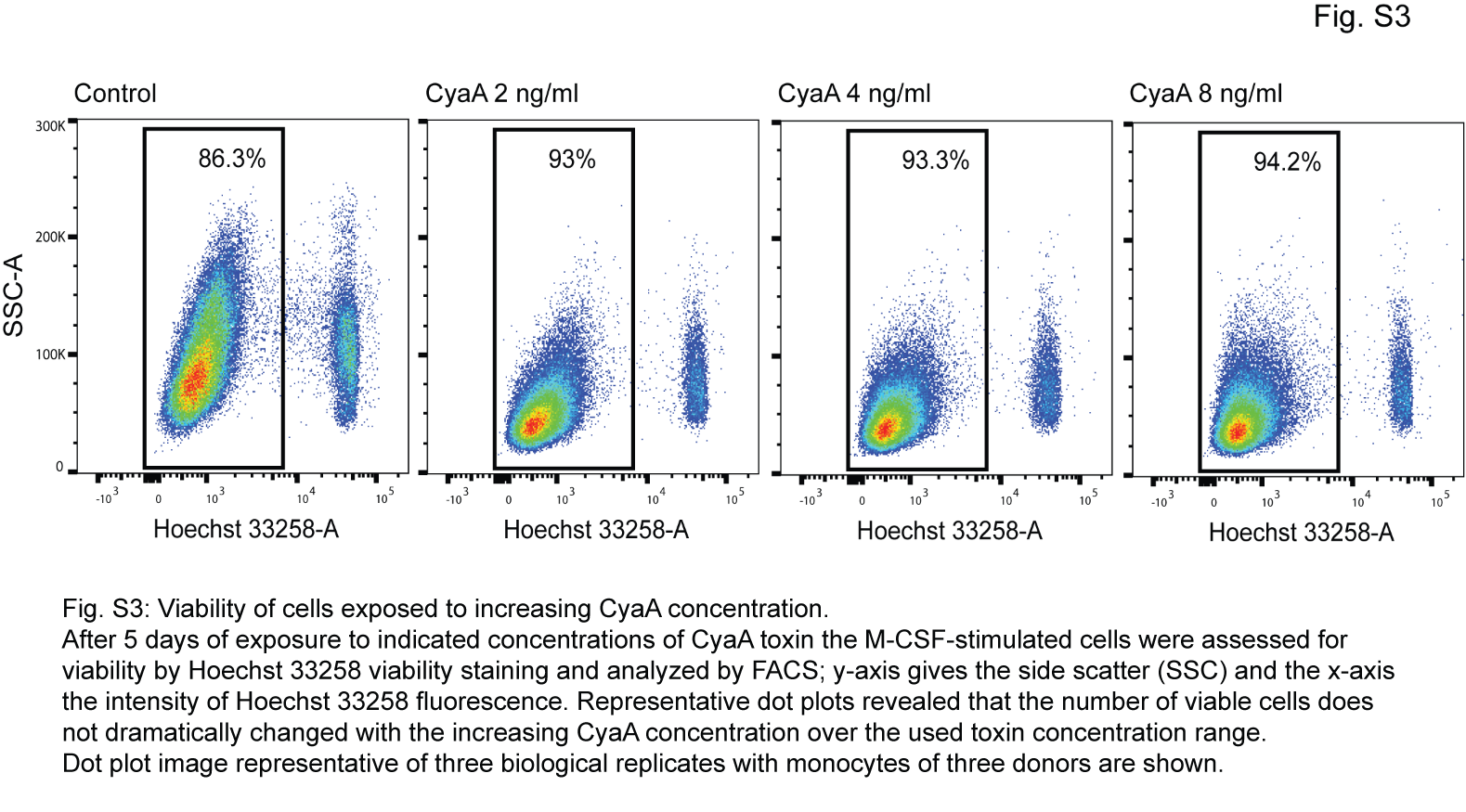

Supplement: Fig. S3 — Viability of cells exposed to increasing CyaA concentration. [file msphere.00407-24-s0003.tif]
