# Supplementary material for: cAMP signaling of Bordetella adenylate cyclase toxin blocks M-CSF triggered upregulation of iron acquisition receptors on differentiating CD14+ monocytes
Source: mSphere. 2024 Jul 30;9(8):e00407-24. doi: 10.1128/msphere.00407-24 (PMC11351043; doi:10.1128/msphere.00407-24)
Supplement: Table S1 — List of primers used for quantification of gene expression by RT-qPCR. [file msphere.00407-24-s0004.docx]

| Gene | Forward primer 5’-3’ | Reverse primer 5’-3’ |
| --- | --- | --- |
| *TFRC (CD71)* | CTGAGTGTGAGAGACTGGCAGG | TCTGTGCTGTCCAGTTTCTCCG |
| *CD163* | GGAGCTGAGGCTAGTGGATGGTG | AGTTGGACATCCCAGCTGGTTAC |
| *HMOX1*  (HO-1) | CAACCCGACAGCATGCCCCA | CCTTGTTGCGCTCAATCTCCTCC |
| *B2M* | GTATGCCTGCCGTGTGAACCATG | CAAATGCGGCATCTTCAAACCTCC |
| *ACTB* | GAAGATCAAGATCATTGCTCCTCCTGAG | GCTGATCCACATCTGCTGGAAGGTG |

**Supplementary table 1. List of primers used for quantification of gene expression by RT- qPCR**
